# Chronopharmacology and Mechanism of Antitumor Effect of Erlotinib in Lewis Tumor-Bearing Mice

**DOI:** 10.1371/journal.pone.0101720

**Published:** 2014-07-07

**Authors:** Peipei Wang, Fengmei An, Xingjun Zhuang, Jiao Liu, Liyan Zhao, Bin Zhang, Liang Liu, Pingping Lin, Mingchun Li

**Affiliations:** 1 Department of Pharmacology, Medical College of Qingdao University, Qingdao, China; 2 Hand Surgery Center of the Whole Army, No. 401 Hospital of Chinese People's Liberation Army, Qingdao, China; 3 Department of Oncology, No. 401 Hospital of Chinese People's Liberation Army, Qingdao, China; 4 Department of Pharmacy, No. 401 Hospital of Chinese People's Liberation Army, Qingdao, China; Kent State University, United States of America

## Abstract

The epidermal growth factor receptor (EGFR), a ubiquitously expressed receptor tyrosine kinase, is recognized as a key mediator of tumorigenesis in many human epithelial tumors. Erlotinib is tyrosine kinase inhibitor approved by FDA for use in oncology. It inhibits the intracellular phosphorylation of tyrosine kinase associated with the EGFR to restrain the development of the tumor. To investigate the antitumor effect of erlotinib at different dosing times and the underlying molecular mechanism via the PI3K/AKT pathway, we established a mouse model of Lewis lung cancer xenografts. The tumor-bearing mice were housed four or five per cage under standardized light-dark cycle conditions (light on at 7:00 AM, 500 Lux, off at 7:00 PM, 0 Lux) with food and water provided ad libitum. The mice were observed for quality of life, their body weight and tumor volume measured, and the tumor growth curves drawn. After being bled, the mice were sacrificed by cervical dislocation. The tumor masses were removed at different time points and weighed. The mRNA expression of EGFR, AKT, Cyclin D1 and CDK-4 were assayed by quantitative real-time PCR (qRT-PCR). Protein expression levels of AKT, P-AKT and Cyclin D1 were determined by Western blot analysis. The results suggest that erlotinib has a significant antitumor effect on xenografts of non-small cell lung cancer in mice, and its efficacy and toxicity is dependent on the time of day of administration. Its molecular mechanism of action might be related to the EGFR-AKT-Cyclin D1-CDK-4 pathway which plays a crucial role in the development of pathology. Therefore, our findings suggest that the time of day of administration of Erlotinib may be a clinically important variable.

## Introduction

Most living organisms exhibit behavioral and physiological rhythms with a period of about 24 h, influenced by environmental factors including light, temperature, water and social interaction and serving to synchronize circadian rhythms to the daily rotation of time [1], [2]. Some of these rhythms are controlled by the circadian clock. Recent molecular studies of the circadian clock have revealed that oscillation in the transcription of specific clock genes plays a central role in the generation of 24-h rhythms [3], [4]. Studies have shown that the rhythms of cancer cells differ from those of normal cells [5]. Changing the timing of administration along the 24-h time scale can profoundly improve tumor responses to the treatment and overall survival rates and reduce drug toxicities in cancer patients [6], [7]. Identification of mechanism involved in the diurnal rhythm of drug susceptibility will help to achieve better chronopharmacotherapy for cancer treatment.

Surgery is the major treatment for most malignant tumors, but recurrence and metastasis often occur after the operations. Systemic chemotherapy can control the recurrence and metastasis effectively, improve the life quality and prolong the survival time of the patients with advance cancers. However, the traditional chemotherapy not only kills tumor cells but also damages the normal cells, resulting in bone marrow suppression, liver and kidney dysfunction, gastrointestinal reactions, decreased immune function and other side effects. Fortunately, this problem can be solved by the molecular targeted drugs. Erlotinib Hydrochloride Tablets (Tarceva) is a new small molecular targeting inhibitor, which inhibits the intracellular phosphorylation of tyrosine kinase associated with the epidermal growth factor receptor (EGFR)[8], [9]. It can selectively act on intracellular targets, block EGFR pathway and inhibit the development of tumors, but causes little damage to the normal cells[10], [11]. Erlotinib monotherapy is indicated for treating the patients with locally advanced or metastatic non-small cell lung cancer after failure of at least one prior chemotherapy regimen[12]. The most common adverse reactions are rash and diarrhea. Its efficiency can be increased but its toxicity reduced by administering the drugs when they are most effective and/or tolerated. The mechanism may be related to the dosing time-dependent variations in pharmacokinetics, tumor responsiveness, and host immune responsiveness [13]. However, the exact mechanism has not been clarified yet.

Erlotinib inhibits cell growth through down-regulation of EGFR phosphorylation. It elicits the transcription of various genes through activation of signal transducers and activators of transcription protein. EGFR is overexpressed or constitutively activated in many types of human cancers, associated with a poor prognosis[14]. EGFR activation can be inhibited by small molecule tyrosine kinase inhibitors (TKI), and inhibition of EGFR function has been shown to decrease the growth of several types of human cancer in preclinical researches[15∼18]. It has been reported that AKT, CDK-4 (cyclin dependent kinases, CDKs), and Cyclin D1 are the downstream signaling molecules of EGFR[19], [20]. Upstream signaling molecules EGFR can stimulate phosphorylation of AKT, activate cellular pathways, and promote tumor cell growth, proliferation, invasion and metastasis[21]. AKT enhances the activity of Cyclin D1 to be combined with CDK-4 to regulate the cell cycle. Both the cell study and the vitro study have proven the overexpression of p-AKT in most human tumor tissues[22]. Therefore, we infer that the mechanism of Erlotinib may be related to EGFR-AKT-CDK4-Cyclin D1 signaling pathway.

The purpose of this paper is to investigate the effects of erlotinib on the inhibition of tumor growth at different dosing times in mice and the underlying mechanism. We aim to find an appropriate time for the chemotherapy to provide the reference to the clinical treatment.

## Materials and Methods

### Animals and Cells

C57BL/6 mice (5 weeks old) were purchased from Vital River Laboratory Animal Technology Co. Ltd. The production license number was SCXK (jing) 2012-0001. The mice were housed four or five per cage under standardized light-dark cycle conditions (light on at 7:00 AM, 500 Lux, off at 7:00 PM, 0 Lux) at (23±1)°C and (50±10)% humidity with food and water provided ad libitum. This study was carried out in strict accordance with the recommendations in the Guide for the Care and Use of Laboratory Animals of the National Institutes of Health. The experiments were approved by the Committee on the Ethics of Animal Experiments of the No. 401 Hospital of Chinese People's Liberation Army.

Lewis lung cancer cells (ATCC CRL-1642) were provided by Beijing Chuanglian North Carolina Biotechnology Research Institute, and maintained in vitro in high glucose DMEM medium supplemented with 10% heated inactivated fetal bovine serum, 0.5% penicillin, and 0.5% streptomycin at 37°C in a humidified atmosphere with 5% CO_2_.

### Drugs and Chemicals

Erlotinib Hydrochloride Tablets (150 mg erlotinib in each tablet) were provided by Roche Ltd. Due to their insolubility in water, they were made into suspension with 0.5% sodium carboxymethyl cellulose. FBS, Trypsin enzyme and high glucose DMEM medium were purchased from HyClone. mRNA extraction kit, cDNA extraction kit, RNA amplification kit, primer design and synthesis were provided by Takara. Protein antibody was purchased from Cell Signaling.

### Tumor Model

The growing cells were collected exponentially and the cell density adjusted. 0.2 ml of 1×10^7^/ml viable tumor cells were inoculated into the subcutaneous of the left hind. Seven days after the tumor cell implantation, the mice were used as tumor-bearing models. They were randomly divided into groups, when the tumors grew to 0.5–1.5 cm^3^.

### Experiment Design

The experiment was performed in a total of 240 female C57BL/6 tumor-bearing mice and 60 normal mice. The tumor-bearing mice were randomly divided into three treatment groups (15, 30, 60 mg·kg^−1^) and one model group. The mice in the treatment groups were administered successively once a day for twenty days by gavage with 15 mg·kg^−1^, 30 mg·kg^−1^, 60 mg·kg^−1^ of erlotinib suspension, respectively. Those in the model group received the same volume of sodium carboxymethyl cellulose.

We selected the 60 mg·kg^−1^ group to investigate the effects of dosing-times on the anti-tumor effects of erlotinib based on the results of the preliminary experiments. The group was randomly divided into 6 time groups (group 8:00, 12:00, 16:00, 20:00, 24:00, and 04:00). The mice in the 6 time groups were administered successively once a day for twenty days via gavage a single dose of erlotinib (60 mg·kg^−1^) at different circadian times: 8:00, 12:00, 16:00, 20:00, 24:00, and 04:00. Those in the model group received the same volume of sodium carboxymethyl cellulose.

### Determination of Antitumor Effect

Diet, exercise and mental status of the mice were observed during the experiment. Tumor volume was measured with calipers every four days and estimated with the formula: tumor volume (cm^3^)  = a^2^×b/2, where a is the shortest diameter, and b is the longest diameter. The antitumor effect of erlotinib was expressed as the tumor volume change. The tumor growth curves were drawn with the data of tumor volume changes. The mice in the 60 mg/kg group were then sacrificed by cervical dislocation at the corresponding experiment times (8:00, 12:00, 16:00, 20:00, 24:00, and 04:00), and samples of tumor mass were removed at different times and weighed. The tumor inhibition rate was calculated using the formula: tumor inhibition rate (%)  =  (mean tumor weight of control group - mean tumor weight of experiment group)/mean tumor weight of control group×100%. The tumor masses were immediately stored in liquid nitrogen for the next experiment.

### Histopathology Analysis

Three tumor masses were collected from each group and fixed in 10% formalin over night. The fixed tumor masses were washed with flowing water for at least 8 hours, then cut into 1.5 cm×1.5 cm×0.2–0.3 cm, and dehydrated with 70% ethanol, 80% ethanol, 90% ethanol, and 100% ethanol. The tumor masses were put into xylene solution for 40 min until they became transparent. Then they were put into 56°C–58°C paraffin, dipped into melted solid paraffin, and made into wax blocks after being fixed. The fixed masses were cut into 4–6 µm, and placed in a chamber at 60°C for 15–30 min to remove the interstitial paraffins. Images were obtained with Leica TCS SP5X by hematoxylin-eosin (HE) staining.

### qRT-PCR Analysis

50 mg frozen tissue was immediately transferred into a mortar, into which liquid nitrogen was added, and crushed with pestle to homogenize until powdery. RNAiso Plus was added according to the amount of homogenized tissue. Chloroform was added to the homogenate solution, mixed well, and then centrifuged to separate the solution into three layers. The top liquid layer was removed into a new tube. An isopropanol precipitation was performed to extract the total RNA, which was reversely transcribed into cDNA according to the instruction of PrineScript RT reagent Kit with gDNA Eraser. The expressions of EGFR, AKT1, CDK-4 and CyclinD1 in tumor tissue were detected by qRT-PCR according to the instruction of SYBR PrimeScript RT reagent Kit. GAPDH primer: F: 5′-TGTGTCCGTCGTGGATCTGA-3′, R: 5′-TTGCTGTTGAAGTCGCAGGAG-3′, 150 bp. EGFR primer: F: 5′-CCTCCACTGTCCAGCTCATTAC-3′, R: 5′-TTCCAGGTAGTTCATGCCCTTT-3′, 140 bp. AKT1 primer: F: 5′-TGAGGTTGCCCACACGCTTA-3′, R: 5′-CCCGTTGGCATACTCCATGAC-3′, 127 bp. CDK-4 primer: F: 5′-CAGAGCTCTTAGCCGAGCGTA-3′, R: 5′- GGCACCGACACCAATTTCAG-3′, 87 bp. CyclinD1 primer: F: 5′-TACCGCACAACGCACTTTC-3′, R: 5′-AAGGGCTTCAATCTGTTCCTG-3′, 84 bp. Reaction parameters were: 95°C denaturation 30 s, 95°C denaturation 5 s, 55°C annealing 30 s, 72°C extension 30 s, 40 cycles. Each sample was repeated for three times and the mean Ct was calculated. The gene expression was estimated with the formula: ΔΔCt  =  (Target gene Ct of experimental group - Reference gene Ct of experimental group) - (Target gene Ct of control group - Reference gene Ct of control group). The relative changes in target gene in different treatment groups were determined by the formula 2^−ΔΔCt^.

### Western-blot Analysis

The frozen tumor masses were transferred into a mortar, into which liquid nitrogen was added, and crushed with pestle to homogenize until powdery. According to the amount of tissue powder, appropriate amount of ice-cold lysis buffer (50 mM Tris–HCl, pH 7.8, 150 mM NaCl, 5 mM EDTA, 0.5% Nonidet P-40, 2 mM PMSF, 1 mM Na_3_VO_4_) was added, and then the homogeneous tissue was cultured on ice for 30 minutes. After the removal of the insoluble materials by centrifugation at 12,000 g for 15 min at 4°C, the resulting supernatants were mixed with an 1/5 volume of 5×sample buffer and boiled at 95°C for 5 min. The protein concentrations in the tumor mass lysates were determined using the BCA protein assay kit (CWBIO, China). The lysate samples were separated on SDS-polyacrylamide gels electrophoresis, and transferred onto a polyvinylidene difluoride (PVDF) membrane (Millipore, US). The membranes were reacted with antibodies against phosphorylated or nonphosphorylated AKT, P-AKT or CyclinD1 (Cell Signaling Technology, US). Thereafter, specific antigen/antibody complexes were made visible using horseradish peroxidase-conjugated secondary antibodies (Rabbit IgG, Cell Signaling Technology, US) and Immobilon Western Chemiluminescent HRP Substrate (Millipore, US). The images from the immune reaction membrane were digitized. The band intensity of each protein was quantified using NIH Image software.

### Statistical Analysis

All data were represented with mean (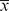) ± standard deviation(SD). The statistical significance of the differences among groups was analyzed by one-way ANOVA and SLD (Least-significant difference) with SPSS 17.0. The 5% level of probability was considered to be significant.

## Results

### Dose-response of erlotinib on tumor growth

The effects of various dosages (15, 30, 60 mg·kg^−1^) of erlotinib on tumor growth in tumor-bearing mice gavaged with the drug for twenty days are shown in Table 1. Relative tumor growth was expressed as the tumor volume growth change from the initiation of erlotinib or odium carboxymethyl cellulose treatment. Tumor growth after initiation of erlotinib treatment was significantly suppressed compared with that in the model group given sodium carboxymethyl cellulose (*P*<0.05). The tumor growth of the 30 mg·kg^−1^ and 60 mg·kg^−1^ groups was significantly different from that of the 15 mg·kg^−1^ group. However, no significant difference of tumor growth was found between 30 mg·kg^−1^ and 60 mg·kg^−1^ groups.

**Table 1:** Dose-response effects of erlotinib on tumor growth (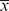
±s, n = 60,N = 240).

Erlotinib dose (mg·kg^−1^)	Tumor volume growth (cm^3^)
Model	4274.83±30.57
15	3183.12±33.15*
30	2183.16±34.74* ^Δ^
60	2074.66±29.09* ^Δ^

^*^P<0.05 when compared with the model group, ^Δ^ *P*<0.05 when compared with the 15 mg·kg^−1^ group.

### Influence of dosing times on the antitumor effect of erlotinib

**Figure 1:**
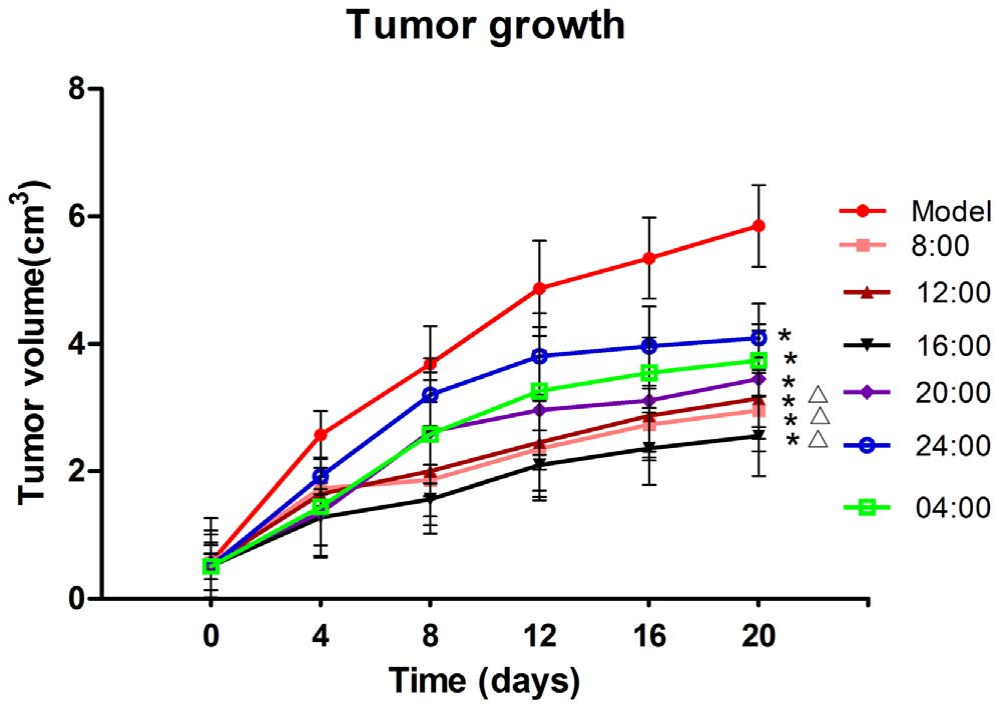
Influence of dosing times on tumor growth after administration of erlotinib or distilled water on three weeks. Each value is the mean with SD of ten mice.^*^ *P*<0.05 when compared with the model group, ^Δ^ *P*<0.05 when compared with groups 20:00, 24:00, 04:00.

Dosing times showed no significant effect on tumor growth in tumor-bearing mice of the model group (data not shown). Therefore, a mean value from different circadian times was used as the control. The tumor growth after erlotinib treatment (60 mg·kg^−1^) at different times was significantly suppressed in the tumor-bearing mice when compared with that in the model mice given sodium carboxymethyl cellulose (*P*<0.05, Figure 1). Tumor growth in groups 8:00, 12:00, and 16:00 in the light phase was significantly suppressed when compared with that in the dark phase (groups 20:00, 24:00, 04:00), with the effect in group 16:00 being the most effective (*P*<0.05). The tumor weights of group 8:00, 12:00, 16:00, 20:00, 04:00 was significantly suppressed when compared with the model (*P*<0.05, Table 2), and group 16:00 showed the best result.

**Table 2:** Tumor weight and inhibition rate of each group (n = 10).

Group	Tumor weight ( 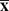 ±s, g)	Inhibition rate(%)
Model	3.93±1.01	*-*
8:00	2.32±0.68*	39.58
12:00	2.61±0.54*	32.03
16:00	1.96±0.77* ^Δ^	48.95
20:00	2.93±0.82*	23.70
24:00	3.17±0.51	17.45
04:00	2.82±0.45*	26.56

^*^ *P*<0.05 when compared with the model group, ^Δ^ *P*<0.05 when compared with group 24:00.

### Influence of dosing times on histopathology

**Figure 2:**
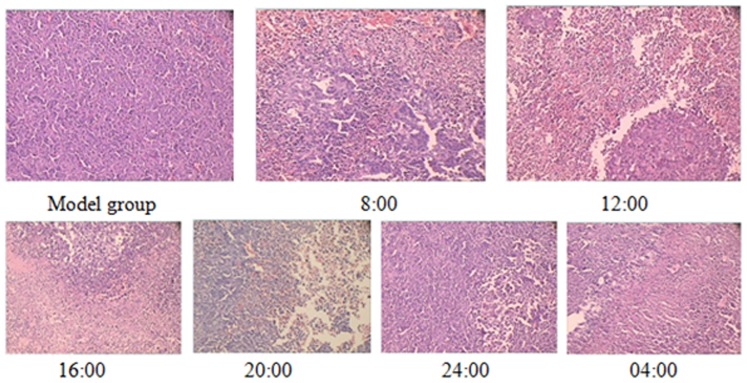
Microscopic images of pathological observation of tumors formed three weeks after the inoculation of lewis lung carcinoma cells into C57BL/6 mice (HE staining, original magnification ×200). (Model group): Pathological section from the model group treated with distilled water. The tumor cells were poorly differentiated and arranged tightly, with abundant vessels around them. No obvious tumor cell necrosis could be observed and the boundary was extremely clear. (Groups 8:00, 12:00, 16:00, 20:00): Pathological section from the groups 8:00, 12:00, 16:00 and 20:00 after erlotinib administration. The tumor cells were poorly differentiated and arranged irregularly, with few new vessels around them. Large areas of necrosis, and inflammatory cell infiltration and bleeding were observed. (Groups 24:00 and 04:00): Pathological section from the groups 24:00 and 04:00 given erlotinib at 24:00 and 04:00. Small focal necrosis and inflammatory cell infiltration were observed.

The photographs in Figure 2 show the representative images about sections of tumor tissues, which display significant differences among different time groups. In the model group, the tumor cells were poorly differentiated and arranged closely. No obvious tumor cell necrosis was observed and the boundary was extremely clear. Large areas of necrosis, and inflammatory cell infiltration and bleeding were observed in groups 8:00, 12:00, 16:00, 20:00 and the tumor cells were poorly differentiated and arranged irregularly, with few new vessels around them. In groups 24:00 and 04:00, small focal necrosis and inflammatory cell infiltration were observed.

### Influence of dosing times on the expression of genes in tumor masses

**Figure 3:**
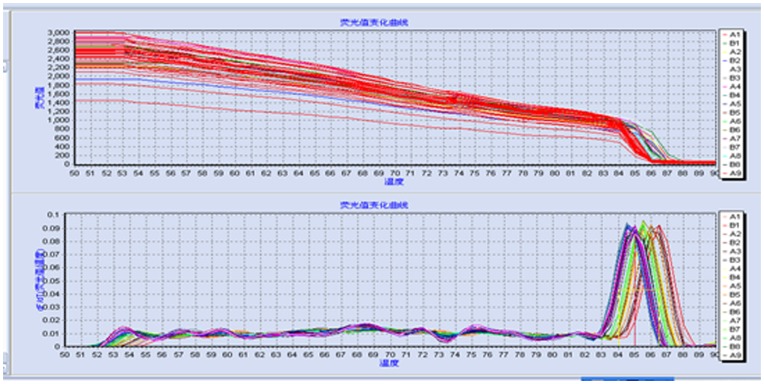
Dissolution curve of gene expression with qRT-PCR. There was only one single peak in dissolution curve and it conforms to the annealing temperature. The results of experiment were effective.

**Figure 4:**
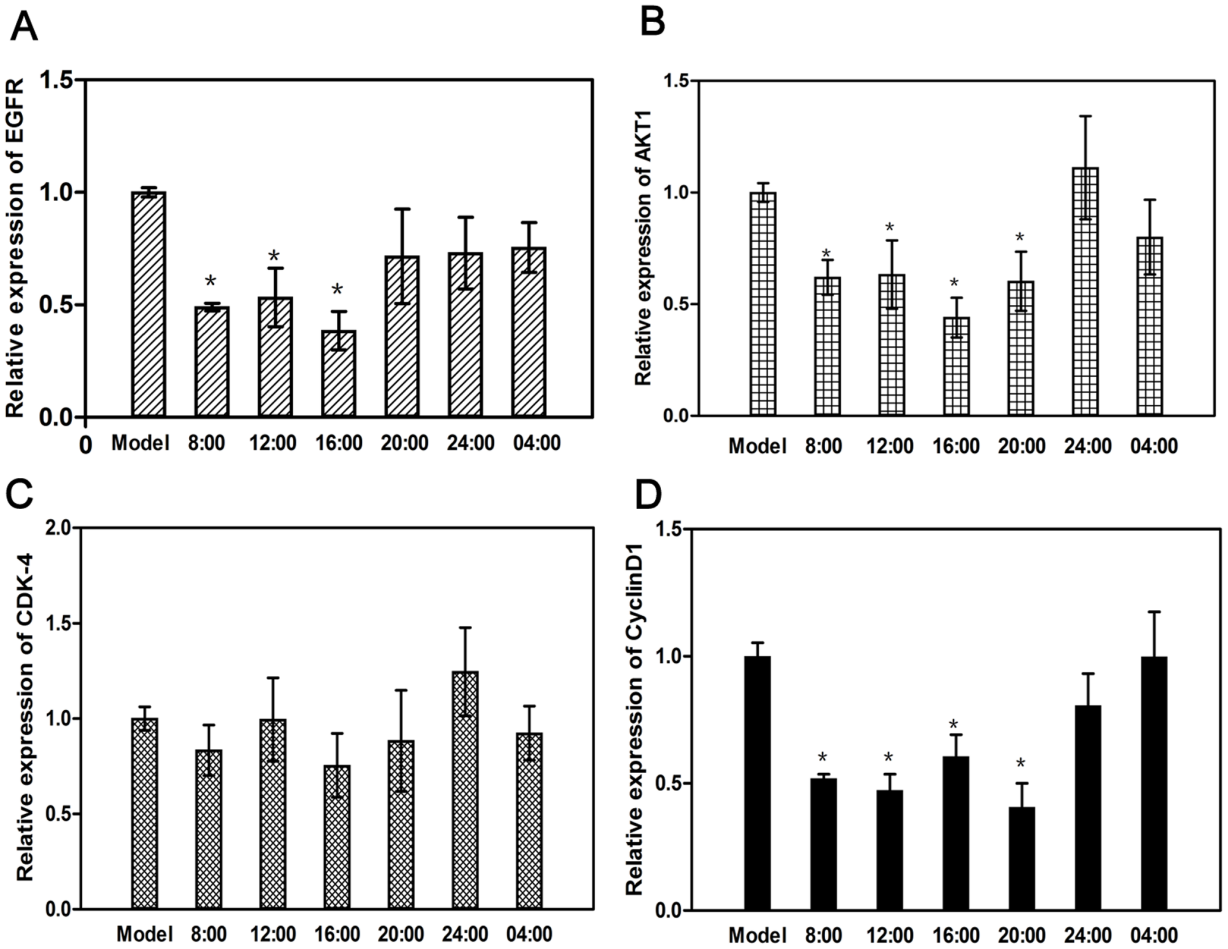
Relative quantitive expression of EGFR, AKT1, CDK-4, and Cyclin D1 mRNA in the tumors from experiment groups (60 mg/kg) and model group (distilled water). Each value is the mean with SD of six mice. (A): The mRNA expression of EGFR in tumors. ^*^ *P*<0.05 vs model group. (B): The mRNA expression of AKT1 in tumors. ^*^ *P*<0.05 vs model group. (C): The mRNA expression of CDK-4 in tumors. There was no significantly different among these groups. (D): The mRNA expression of Cyclin D1 in tumors. ^*^ *P*<0.05 vs model group.

There was only one single peak in the dissolution curve conforming to the annealing temperature (Figure 3), which shows that the results of our experiment were effective. As shown in Figure 4, the expression of EGFR in groups 8:00, 12:00, 16:00 was significantly lower than that of the model group (*P*<0.05), and that of group 20:00, 24:00, 04:00 had no significant change when compared with the model group (*P*>0.05). The expression of AKT1 in groups 8:00, 12:00, 16:00 and 20:00 was significantly lower than that in the model group (*P*<0.05), the group 16:00 showed the best result (*P*<0.05), and that of groups 24:00 and 04:00 had no significant change when compared with the model group (*P*>0.05). The expression of CDK-4 in all groups was not significantly lower than that in the model group (*P*>0.05). The expression of CyclinD1 in groups 8:00, 12:00, 16:00 and 20:00 was significantly lower when compared with that of the model group (*P*<0.05), and that of groups 24:00 and 04:00 had no significant change when compared with the model group (*P*>0.05).

### Influence of erlotinib dosing time on AKT, P-AKT, and Cyclin D1 protein levels in tumor masses

**Figure 5:**
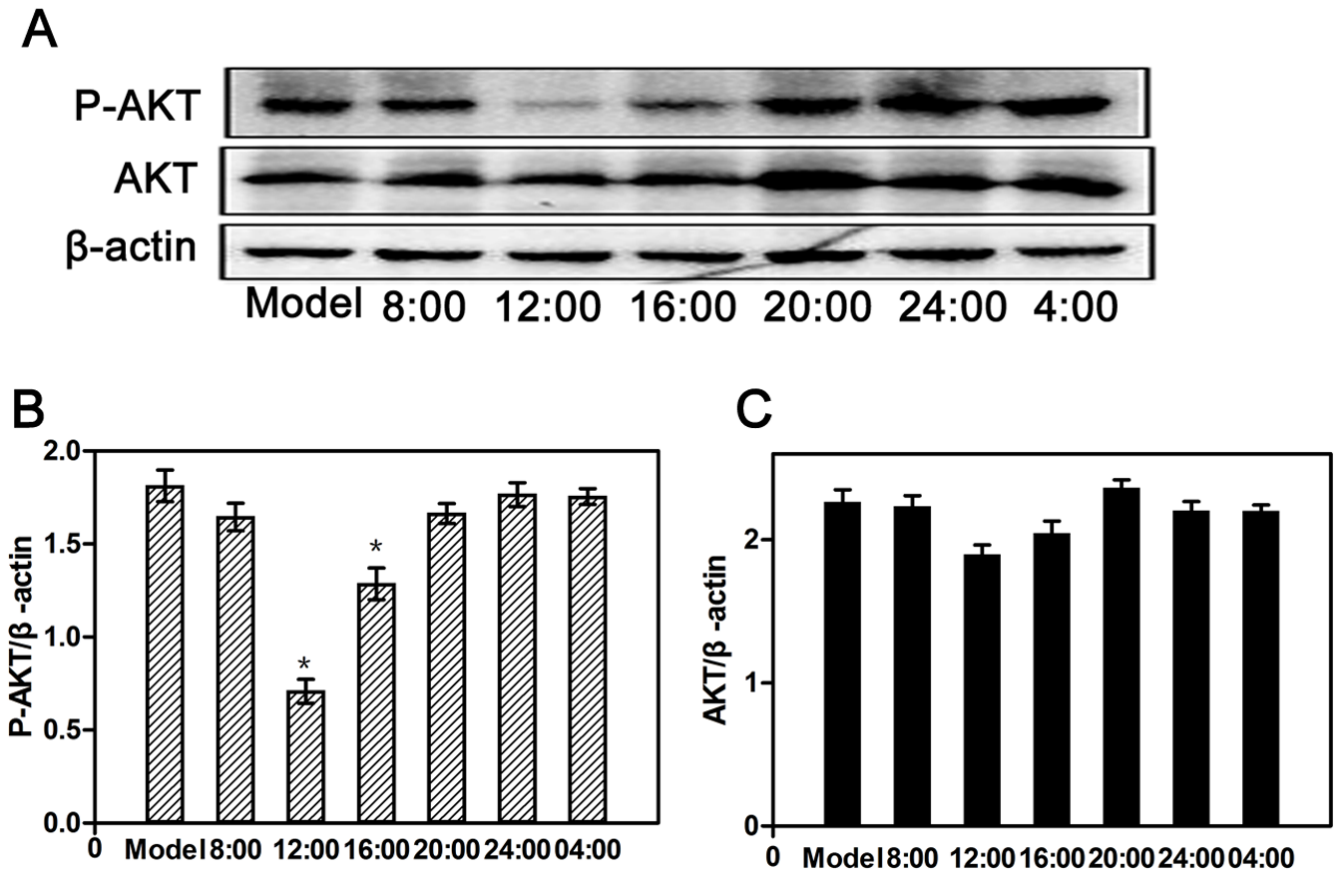
Influence of dosing times on P-AKT and AKT protein expression (A) or relative P-AKT and AKT protein expression (B and C) in tumor masses after erlotinib (60 mg/kg) administration. Each value is the mean with SD of six mice. ^*^ *P*<0.05 when compared with the model group.

**Figure 6:**
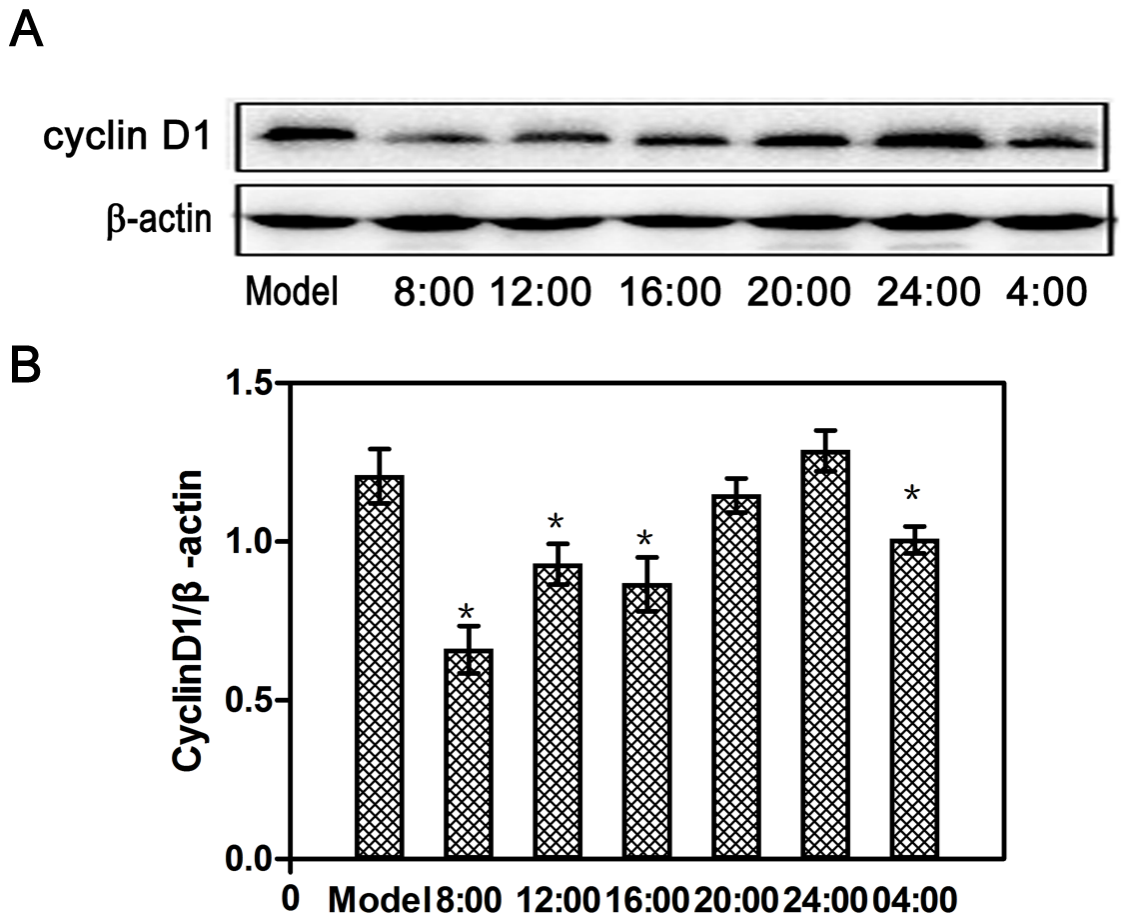
Influence of dosing times on Cyclin D1 protein expression (A) or relative CyclinD1 protein expression (B) in tumor masses after erlotinib (60 mg/kg) administration. Each value is the mean with SD of six mice. ^*^ *P*<0.05 when compared with the model group.

As shown in Figure 5, the P-AKT protein level in groups 12:00 and 16:00 was significantly lower than that in the model group (*P*<0.05), and it was significantly different between groups 12:00 and 16:00, while the level of AKT remained unchanged (*P*>0.05). As shown in Figure 6, the Cyclin D1 protein level in groups 8:00, 12:00 and 16:00 and 04:00 was significantly lower than that in the model group (*P*<0.05).

## Discussion

Chronochemotherapy, as a new form of chemotherapy, has developed rapidly in the clinical treatment of tumors. It is based on the circadian rhythm of tumor cell synthesis, the related protein factors of drug targets and living organisms themselves. The relationship between the circadian rhythm in drug tolerability and antitumor efficacy constitutes an essential issue for cancer chronotherapy. Studies have shown that chronochemotherapy can significantly prolong the overall survival of cancer patients when compared with conventional chemotherapy and its toxicity can be controlled[23]. Recently, the best times of administration of about 30 drugs have been found, including 5-fluorouracil, methotrexate, vinorelbine, etc [24], . However, the study on chronopharmacology of molecular targeted drugs has not been reported. As a small molecular-targeted drug, erlotinib has been used for the treatment of advanced NSCLC. Its clinical efficacy has been proved by researches, especially of cancer-related genes and proteins. Erlotinib is effective in treating NSCLC because it can reversibly and competitively inhibits the binding of ATP to the phosphate-binding loop of the ATP site in the intracellular domain of EGFR. By inhibiting the binding of ATP to EGFR, the drug restrains auto-phosphorylation and the activation of downstream signaling pathway further, leading to the inhibition of cell proliferation and inducing apoptosis in NSCLC. Therefore, we chose erlotinib to study, and found that the antitumor effect of erlotinib showed circadian rhythm in our preliminary experiments.

The division, proliferation, and metabolism of cells are related to biological circadian rhythm. Studies[27], [28] show that proliferating cells are the most sensitive to anticancer drugs, and DNA synthesis usually peaks between noon and 16:00 and down to the bottom at midnight. Therefore, we selected six hour points, 8:00, 12:00, 16:00 (as the light phase), 20:00, 24:00, 04:00 (as the dark phase), according to the circadian rhythm of DNA synthesis, mouse circadian rhythms and references. Based on the results of dose conversion between human and animals and the preliminary experiments, we selected the doses of 15, 30, and 60 mg·kg^-1^ in our experiment. We investigated the influence of dosing times on the effects of erlotinib to inhibit tumor growth in mice and the underlying mechanism. The results suggested that the antitumor effect of erlotinib showed a significant circadian rhythm with higher levels in the light phase, and the group 16:00 showed the best result. On the contrary, the toxicity of erlotinib showed a significant circadian rhythm with higher levels in the dark phase, especially in the groups 24:00 and 04:00. Generally speaking, the administration of erlotinib in the light phase may be more effective than in the dark phase, which may be related to the different sensitivity of cells to antitumor drugs in different periods.

Until now the mechanism of chronochemotherapy of erlotinib remains unclear. Recent advances identify critical molecular events including that drug metabolism and detoxification controlled by biological rhythms, cell cycle, molecular targets, DNA repair, apoptosis, and angiogenesis. It may be related to drug metabolism, some enzymes of cell cycle or some factors associated with cell signaling pathways[29]. The target of erlotinib is EGFR. Erlotinib inhibits tumor growth by inhibiting EGFR autophosphorylation to block its downstream signal transduction. AKT, CDK-4, and CyclinD1 are the downstream signaling factors of EGFR signaling pathway. Some studies[30] have shown that EGFR plays an important role in angiogenesis, tumor cell metastasis and apoptosis. Based on these findings, we investigated whether the EGFR signaling network was sensitive to the small molecule TKI erlotinib. CyclinD1, G1 phase cyclin, is regulated by growth factors in the cell cycle. It can be combined with CDK4 or CDK6 to form complexes to promote cell proliferation, and lead to tumors when CyclinDl is expressed out of control[31]. In this study, the expression of genes EGFR, AKT, CDK-4, and CyclinD1 and the proteins AKT, p-AKT and CyclinD1 were found to show circadian rhythm on different dosing times. The expressions of these genes or proteins in the light were significantly lower when compared with the model group. It shows that erlotinib can effectively inhibit EGFR signaling through the AKT pathways. Therefore, we can conclude that the mechanism of chronochemotherapy of erlotinib may be related to the apoptosis pathway mediated by EGFR-AKT-CyclinD1-CDK-4 pathway.

This study suggests that the dosing time-dependent change in the antitumor activity of erlotinib is caused by that in the sensitivity of tumor cells and the circadian rhythm of organisms. Furthermore, the time-dependent changes in the sensitivity of tumor cells may be related to the EGFR signaling pathway. In conclusion, the choice of dosing time based on the diurnal rhythm may help to establish a rational chronotherapeutic strategy, increasing the antitumor activity of the drug in certain clinical situations.

This paper may be not perfect for some practical difficulties in the experiment, so further studies on specific and thorough molecular mechanism will be performed in our further study.
